# Computer‐assisted revision total knee arthroplasty does not improve postoperative knee prosthesis alignment compared to the conventional technique

**DOI:** 10.1002/jeo2.12064

**Published:** 2024-07-16

**Authors:** Triine E. Alling, Marrigje F. Conteh‐Meijer, Alexander L. Boerboom, Martin Stevens, Inge H. F. Reininga

**Affiliations:** ^1^ Department of Orthopaedics, University Medical Center Groningen University of Groningen Groningen The Netherlands; ^2^ Department of Orthopaedics Martini Hospital Groningen Groningen The Netherlands; ^3^ Department of Trauma Surgery, University Medical Center Groningen University of Groningen Groningen The Netherlands

**Keywords:** alignment, computer‐assisted surgery, knee arthroplasty, revision surgery, surgical technique

## Abstract

**Purpose:**

Computer‐assisted surgery (CAS) during primary total knee arthroplasty (TKA) prosthesis alignment. However, literature on its use during revision TKA (rTKA) is scarce. Moreover, the effect of CAS during rTKA on rotational alignment of the prosthesis has not been described yet. The purpose of this study was to assess the effect of CAS during rTKA, focusing on the number of outliers and coronal, sagittal and rotational prosthetic alignment compared to conventional rTKA.

**Methods:**

A prospective cohort study comparing CAS‐rTKA with a historical control group (CON‐rTKA). The CAS‐rTKA group (54 patients/62 knees) underwent rTKA using imageless CAS between 2012 and 2017. The CON‐rTKA group (13 patients/23 knees) was operated using the conventional technique between 2002 and 2012. Postoperative alignment was measured using the EOS‐2D/3D system (coronal and sagittal planes) and computed tomography scan (rotation).

**Results:**

No significant differences between the CAS‐rTKA and CON‐rTKA groups were found for coronal and sagittal alignment regarding the mechanical angle of the leg (*p* = 0.08), mechanical lateral distal femoral angle (*p* = 0.87), mechanical medial proximal tibial angle (*p* = 0.40), anatomical proximal posterior tibial angle (*p* = 0.43) nor femoral (*p* = 0.80) and tibial rotation (*p* = 0.15). For the proportions of coronal, sagittal and rotational outliers, no significant differences were found either.

**Conclusion:**

This study showed no evidence that use of CAS during rTKA leads to improved coronal, sagittal or rotational alignment of knee prostheses or a difference of outliers between the groups.

**Level of Evidence:**

Level III, therapeutic.

**Abbreviations**
AORI: Anderson Orthopaedic Research Institute
aPPTA: anatomical proximal posterior tibial angle
CAS: computer‐assisted surgery
CAS‐rTKA: intervention group on which computer‐assisted surgery is used during rTKA
CON‐rTKA: historical control group on which the conventional technique is used during rTKA
EOS: electro optical system
GCT: geometric centre of the tibia
HKA: mechanical angle of the leg
LCCK: Legacy Constrained Condylar Knee
mLDFA: mechanical lateral distal femoral angle
mMPTDA: mechanical medial proximal tibial angle
rTKA: revision total knee arthroplasty
TCA: tibial component axis
TKA: total knee arthroplasty

## INTRODUCTION

The main challenge in revision surgery is restoration of joint stability, which can be complicated due to loss of bone stock and difficulties identifying relevant bony landmarks secondary to primary surgery [30]. Achieving optimal prosthetic alignment during revision total knee arthroplast (rTKA) is essential, as malalignment can lead to uneven load distribution and suboptimal restoration of joint biomechanics [35]. This has been linked to early (aseptic) loosening of the knee prosthesis and is, after infection, the second‐most frequent mode of failure of primary knee prostheses [1]. Computer‐assisted surgery (CAS) has been developed to improve the accuracy of prosthetic component placement by providing real‐time feedback on resection parameters based on tibial and femoral cutting guides. This helps correct alignment to ensure that optimal cut parameters are achieved, leading to improvements in component and limb alignment [35].

Multiple studies show improved coronal and sagittal postoperative prosthesis alignment after primary TKA using CAS, compared to conventional TKA [2, 6, 10, 13, 16, 28, 36]. Studies on the application of CAS in rTKA, however, are scarce due to the more complicated surgical technique and novelty of CAS during revision surgery. Therefore, whether CAS also improves alignment in rTKA (CAS‐rTKA) remains under debate [30]. Meijer et al. performed a systematic review that gave an overview of the existing literature on this topic. They concluded that evidence is scarce and that almost all studies were underpowered when comparing rotational alignment. A retrospective study by Jenny and Diesinger [18] compared postoperative mechanical alignment of 50 CAS‐rTKAs with 36 conventional rTKAs, finding a significant improvement in alignment using CAS and concluding its superiority in assuring accurate prosthetic alignment. Perlick et al. [30] compared the results of 25 CAS‐rTKAs to 25 conventional rTKAs, demonstrating a significantly better mechanical limb axis plus superior coronal orientation of the femoral component with fewer outliers when using CAS compared to the conventional technique. Thus, a few studies do indicate that CAS improves prosthetic alignment in rTKA. However, study groups are small and only two studies [18, 30] compared the results with conventional rTKA. No studies have yet assessed the effect of CAS on rotational alignment during rTKA. The aim of this study was therefore to assess the effect of CAS during rTKA, focusing on coronal, sagittal and rotational prosthetic alignment.

We hypothesize that using CAS during rTKA will lead to improved prosthesis alignment and less outliers compared to using mechanical alignment guides.

## MATERIALS AND METHODS

### Study design

A prospective cohort study using a historical control group was conducted at the Department of Orthopaedics of University Medical Center Groningen. The intervention group consisted of patients who underwent rTKA between January 2012 and January 2017 using an imageless CAS system. The historical control group consisted of patients who underwent rTKA without use of CAS between January 2002 and January 2012. The study design is extensively described elsewhere [28].

### Study population

Inclusion criteria were:

– Total or partial revision of either the tibial or femoral part of the total knee prosthesis. For partial revisions, only measurements of the part of the prosthesis to be revised were used.
– Use of imageless CAS during rTKA for the intervention group and use of conventional alignment guides during rTKA for the control group.
– Minimum age 18 years.

Exclusion criteria were:

– Minor revision with only replacement of inserts or patella resurfacing.
– Placement of a tumour prosthesis during rTKA.
– Placement of a rotating hinge knee prosthesis during rTKA.
– Revision because of an infected knee prosthesis.
– Limited knowledge of the Dutch language or limited mental capability to participate in the study.

The anaesthetic, analgesic and postoperative physiotherapy protocols were identical for both groups. Demographic characteristics, body mass index, indication for surgery, type of revisions (total/partial femur/partial tibia), operation time and length of hospital stay were collected and/or recorded for all patients included in this study.

### rTKA surgical procedure

The surgical procedure for both groups consisted of three main steps: (1) implant removal, (2) classification of defects and (3) rebuilding of the knee joint by preparing the tibia first. The first step was to extract the failing implant components and to remove all debris. As much bone as possible was preserved, bone of poor quality was removed in the process. The second step was to categorize the bone defects according to the Anderson Orthopaedic Research Institute (AORI) classification [14]. In defect types 2A, 2B and 3, bone loss was compensated by metal augmentations, while stems attached to the tibial and femoral components provided for alignment and diaphyseal load transfer. The third step was rebuilding the knee joint with a revision prosthesis. The NexGen® Legacy Constrained Condylar Knee® revision system (Zimmer Inc.) was used at the Department of Orthopaedics of the hospital where the research was performed. Tibial and femoral revision components were placed with cemented stems, and, if needed, with augmentation blocks or trabecular metal cones. The prosthesis was fixed with bone cement (Refobacin® revision bone cement with clindamycin and gentamicin; Biomet Inc.). Type of articulating surface (posterior stabilized or condylar constrained) was chosen during surgery depending on the stability and integrity of the collateral ligaments.

### Intervention group

In the intervention group, imageless CAS was implemented during rTKA. The ORTHOsoft Navitrack® navigation system (Zimmer Inc.) was used. The navigation is based on an infrared reflecting system with use of trackers on the femur and tibia. The system guides the surgeon by an image‐free model based on anatomical landmarks identified by the surgeon. After exposure, a femoral tracker was placed proximally from the knee in the same knee wound. The tibial tracker was placed in an additional 3‐cm incision above the ankle. Before removing the primary prosthesis, the navigation protocol was applied in which the anatomical landmarks were chosen, and the system built its model from the patient's data. All anatomical landmarks were thus identified with the primary prosthesis in situ. The mechanical axis of the lower limb was the angle between the mechanical axis of the femur and tibia and was measured with the navigation system. The mechanical axis of the femur was the axis between the centre of the femoral head and the distal entry point of the medullary canal. The centre of the femoral head was determined by moving the leg in a conical pattern, digitizing 14 distinct positions of the femoral tracker. The entry point of the distal medullary canal was marked by the orthopaedic surgeon. The mechanical axis of the tibia was the axis between the proximal entry point of the medullary canal and the centre of the ankle. The entry point of the medullary canal was marked by the orthopaedic surgeon, and the centre of the ankle was assessed by marking the medial and lateral malleoli. The coronal and sagittal prosthetic alignment of the femoral and tibial components was calculated according to the femoral and tibial mechanical axes, respectively. Rotation of the femoral component was determined according to the epicondylar axis. The orthopaedic surgeon marked the medial and lateral epicondyles, thereby generating this axis. Rotation of the tibial component was assessed in relation to the axis between the middle of the original insertion of the posterior cruciate ligament and the medial third of the tibial tuberosity. Both bony landmarks were marked by the orthopaedic surgeon. The surgeon was guided by the navigation system to position the components and determine the size of the implants.

### Control group

In the control group, the position of the revision prosthesis was determined using NexGen® mechanical intramedullary alignment guides for the femur and tibia (Zimmer Inc.). Positioning of the components and sizing were based on the same anatomical landmarks as in the intervention group with use of prototypes and trial components.

### Radiographic evaluation of prosthetic alignment

To evaluate alignment in the coronal and sagittal planes, the EOS‐2D/3D system (EOS Imaging) [13] was used. This device reduces radiation by 800–1000 times less than a computed tomography (CT) scan and 10 times less than a conventional X‐ray [13, 19]. The EOS imaging device uses two orthogonal sources of radiation and linear detectors that are coupled together. These sources moved up and down along the patient, producing simultaneous coronal and sagittal images while the patient is in weight‐bearing position. This is a different technique than conventional radiograph systems, where divergent beams in the horizontal and vertical planes allow for both two‐ (2D) and three‐dimensional (3D) images to be taken. EOS images were used to measure prosthetic alignment in the two groups. In case of prostheses on both sides, one leg was placed slightly in front of the other to allow for correct measurements. For both groups, standard coronal and sagittal X‐rays were taken postoperatively in addition to the EOS images, as part of the standard operation protocol. Measurements were taken by one person (M. F. C‐M.) who had extensive experience performing EOS‐2D and 3D measurements before the start of this study.

Angles measured for coronal and sagittal alignment were:

– Mechanical angle of the leg (HKA): Angle between the line from the femoral head to the centre of the knee and the line from the centre of the ankle to the centre of the knee in the coronal plane.
– Mechanical lateral distal femoral angle (mLDFA): Angle between the mechanical axis of the femur and the articular surface of the femoral part of the prosthesis in the coronal plane.
– Mechanical medial proximal tibial angle (mMPTA): Angle between the mechanical axis of the tibia and the articular surface of the tibial part of the prosthesis in the coronal plane.
– Anatomical proximal posterior tibial angle (aPPTA): Angle between the mechanical axis of the tibia and the articular surface of the tibial part of the prosthesis in sagittal plane. The downslope of the design of Nexgen prostheses is 7°; this angle is considered the normal aPPTA.

HKA was measured in 3D using sterEOS software (EOS Imaging) and following the previously described measurement protocol [26]. The mLDFA, mMPTA and aPPTA were measured in 2D, since 3D measurements were not possible with the sterEOS software. For HKA, mLDFA, mMPTA and aPPTA, a generally accepted outlier cut‐off of ±3° was applied in this study [12, 17, 32, 33].

Rotational prosthetic alignment was measured on a CT scan of the operated knee. This CT scan was made postoperatively for the intervention group patients during outpatient clinical follow‐up visits. Patients in the historical control group were invited for an appointment in our hospital, during which a CT scan was made.

To evaluate rotational prosthetic alignment, rotation of the femoral component was determined according to the Berger CT protocol [3, 5]. The CT scans were anonymized by removing names and patient numbers. The measurements were performed blindly by an orthopaedic surgeon (A.L.B.) with extensive experience in performing these measurements. Angles measured for rotational alignment were:

– Condylar twist angle for rotation of the femoral component: angle between the epicondylar axis and the prosthetic posterior condylar axis (inner border of posterior cut). External rotation of the femoral component is shown as a positive (+) angle and internal rotation of the femoral component is shown as a negative (−) angle. An angle >3° internal rotation or external rotation is considered as an outlier [8, 15, 21].
– Rotation of the tibial component: the angle between the tibial component axis and the line connecting the geometric centre of the tibia to the midpoint of the tibial tubercle. Normal rotation using this method is 18° (±2.5°) internal rotation [3]. An angle >3° endorotation or exorotation is considered as an outlier [8, 21, 34].

### Sample size

Our hypothesis was that using CAS leads to fewer outliers in prosthetic alignment than using mechanical alignment guides during rTKA. Primary outcome measure was rotational prosthetic alignment. In surgeries with the standard operation technique, around 25% of knees are considered radiological outliers [11, 23]. Research shows that using CAS in TKA decreases the number of outliers by 17%–30% [11, 20, 24]. We expected a 20% decrease in outliers in the CAS group compared to the historical control group. Hence, with P1 0.95, P2 0.75, *α* 0.05 and power 80%, 44 knees per group were needed. Ultimately, we succeeded in including 23 knees in the control group. A substantial part of the patients initially approached did not meet the inclusion criteria and not everyone was able to take part in the study due to reasons such as physical disability or mental illness. Including patients operated on before January 2002 was not deemed feasible due to the use of a different knee revision system and different surgeons. Still both groups can be considered comparable based on patient demographics and characteristics.

### Statistical analysis

Statistical analyses were performed using IBM SPSS Statistics for Windows software (version 28.0; IBM SPSS). Descriptive statistics were used to describe the main characteristics of the two groups. For the clinical parameters, *t*‐tests were used for continuous values or the Mann–Whitney *U*‐test for non‐normally distributed variables. Differences in rotational, coronal and sagittal alignment between the groups were determined by using the nonparametric Mann–Whitney *U*‐test for independent samples. A *χ* ^2^ test and a Fisher's exact test were used for dichotomous values. For all test procedures, a *p* value <0.05 was considered statistically significant.

### Ethical aspects

Patients were informed that data of their CAS measurements and radiographs could be used for scientific research. The patients included in this study provided written consent to participate in this study and agreed to an extra CT scan postoperatively as approved by the Medical Ethical Review Board; IRB approval was issued by the METc (2012/220). The data of patients who had objections to the use of their data were not included in the study.

## RESULTS

**Figure 1:**
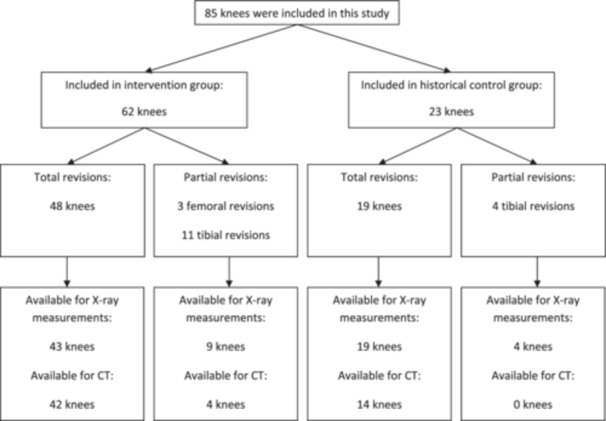
Flow chart of the included patients. CT, computed tomography.

A total of 85 knees (67 patients) were included in this study (Figure 1): 62 knees (54 patients) in the intervention group (CAS‐rTKA) and 23 knees (13 patients) in the historical control group (CON‐rTKA). Five patients were allocated to the CAS‐rTKA group with one knee and to the CON‐rTKA group with the other knee. Patient demographics and characteristics are presented in Table 1 and were equal between groups, aside from the number of inclusions in the CAS‐rTKA and CON‐rTKA groups. Operation time for total and partial revisions and length of hospital stay did not show significant differences between groups (Table 1). Main indications for revision were aseptic loosening (31 cases), malpositioning (24 cases) and instability/wear (14 cases).

**Table 1:** Patient demographics and characteristics.

	CAS‐rTKA	CON‐rTKA	*p* Value
Number of knees (M/F)	62 (29/33)	23 (12/11)	0.67
Number of total revisions (total/partial femur/partial tibia)	48/3/11	19/0/4	0.38
Age^[Link]^, ^a^ (years)	63.8 (9.3)	60.3 (13.7)	0.26
Body mass index^[Link]^, ^a^	30 (4.3)	30 (5.8)	0.94
Operation time total revisions (min)^[Link]^, ^a^	282 (46)	258 (46)	0.06
Operation time partial revisions (min)^[Link]^, ^a^	204 (71)	168 (50)	0.36
Length of hospital stay^[Link]^, ^a^	8 (2.5)	9 (2.5)	0.08

Abbreviations: CAS‐rTKA, intervention group on which computer‐assisted surgery is used during rTKA; CON‐rTKA, historical control group on which the conventional technique is used during rTKA; F, female; M, male; rTKA, total knee arthroplasty.

^a^ Values are given as mean ± standard deviation.

### Alignment measurements

In the CAS‐rTKA group, the HKA could not be measured in nine cases due to a technical error of the software; measurement of the HKA was thus possible in 53 cases. In three cases, a CT was not available for measurements of the medial and/or lateral epicondyle in the CAS‐rTKA group. In six cases, rotation of the tibial component could not be measured because the tuberosity was not visual on CT scan, leaving 56 knees available for evaluation of tibial rotational alignment. In the CON‐rTKA group, the tibial tuberosity could not be assessed on CT‐scan in three cases, leaving 20 knees available for evaluation of tibial rotation (Table 2).

**Table 2:** Postoperative alignment angles for the CAS‐rTKA and CON‐rTKA groups.

Alignment angle	*N*	CAS‐rTKA	*N*	CON‐rTKA	*p* Value
HKA^a^	53	0.4 (3.2)	23	−1.4 (3.8)	0.08
mLDFA^a^	49	0.0 (3.6)	19	0.0 (2.6)	0.97
mMPTA^a^	56	0.0 (2.0)	23	0.0 (2.0)	0.40
aPPTA^b^	56	4.8 (2.8)	23	5.3 (2.1)	0.43
Rotation femur^a^	47	−1.0 (3.0)	17	−0.9 (4.0)	0.80
Internal rotation tibia^b^	56	18.2 (7.5)	20	15.2 (8.1)	0.15

Abbreviations: aPPTA, anatomical proximal posterior tibial angle; CAS‐rTKA, intervention group on which computer‐assisted surgery is used during rTKA; CON‐rTKA, historical control group on which the conventional technique is used during rTKA; HKA, mechanical angle of the leg; IQR, interquartile range; mLDFA, mechanical lateral distal–femoral angle; mMPTA, mechanical medial proximal tibial angle; rTKA, total knee arthroplasty.

^a^ Median and IQR are displayed in degrees.

^b^ Mean ± standard deviation is displayed in degrees.

Coronal and sagittal alignment measurements and femoral and tibial rotation did not differ significantly between groups. The proportions of coronal, sagittal and rotational outliers did not differ significantly between the CAS‐rTKA and the CON‐rTKA group (Table 3).

**Table 3:** Distribution of outliers for different alignment angles for the CAS‐rTKA and CON‐rTKA groups.

Alignment angle	Number of outliers/no outliers CAS‐rTKA	Number of outliers/no outliers CON‐rTKA	*p* Value^[Link]^, ^a^
HKA	12/41	7/16	0.47
mLDFA	6/43	4/15	0.45
mMPTA	7/49	1/22	0.43
aPPTA	19/43	6/17	0.67
Rotation femur	4/43	4/13	0.19
Rotation tibia	30/26	13/7	0.38

Abbreviations: aPPTA, anatomical proximal posterior tibial angle; CAS‐rTKA, intervention group on which computer‐assisted surgery is used during rTKA; CON‐rTKA, historical control group in which the conventional technique is used during rTKA; HKA, mechanical angle of the leg; mLDFA, mechanical lateral distal–femoral angle; mMPTA, mechanical medial proximal tibial angle; rTKA, total knee arthroplasty.

^a^ *χ* ^2^ test.

## DISCUSSION

The most important finding of this study was that there are no differences between coronal and sagittal alignment measurements. No significant differences in the proportions of coronal, sagittal and rotational outliers between groups could be determined either.

One of the most important goals of rTKA is restoration of alignment and CAS has been shown to be a valuable tool in assuring accurate prosthetic component placement. In this study, an imageless CAS system was used. An image‐based system was not considered as this would need a preoperative CT or MRI. However, a CT or MRI of a knee with a prosthesis in situ will be strongly influenced by the presence of the prosthesis and this scattering or magnetic field interaction will most likely disturb the surface matching as needed in image‐based CAS. The restoration of alignment has only been demonstrated in primary surgeries and two studies have compared the results of CAS‐rTKA with conventional methods [18, 30]. In primary TKA procedures, cutting errors during tibial and femoral resection have been linked with adverse effects regarding component alignment [35]. According to Schwarzkopf et al. [35] despite multiple sources of alignment error associated with resection cuts in TKA, CAS can play a valuable role in confirming the resection angles, thereby providing an additional cross‐check against inaccurate cuts. To our knowledge, this study is the first to analyse the effect of CAS on rotational alignment in rTKA. Rotational alignment of the femoral or tibial component was not significantly different between the two groups either. The proportion of outliers for coronal, sagittal or rotational alignment measurements did not differ significantly between groups.

A systematic review by Meijer et al. [27] concluded against current evidence that CAS improves rotational alignment of either the femoral or tibial component or leads to fewer outliers. These conclusions must be interpreted with caution: the number of available studies was low, studies differed in methodological quality, different methods for assessing rotation of the tibial component were used and only one of the included studies based the power analysis on rotational alignment [27]. No gold standard exists for measurement of rotation of the tibial component post‐TKA. References used for measurements include the tip and medial third of the tuberosity [4, 9, 25], ankle [34] and posterior border of the proximal tibia [21]. In this study, the posterior border of the tibial plateau was measured relative to the projection of the tibial tuberosity. It was decided to perform the CT measurements in this way due to the goal of aligning the tibial component relative to the tuberosity during rTKA surgery. However, identifying the tibial tuberosity on the CT scan following rTKA can be challenging and is not always possible, leading to discrepancies in the comparison of outcomes.

Coronal and sagittal measurements were performed using the EOS‐2D/3D stereoradiography system [11]. In this study, the sterEOS software (EOS Imaging) was used for the HKA measurements in 3D, following the measurement protocol as described by Meijer et al. [26]. In 2D, HKA measurements are influenced by the position of the lower limb during acquisition, namely varus/valgus angle and lower limb rotation and flexion [7, 22, 31, 37]. EOS‐3D HKA measurements have proven to be more valid than 2D HKA measurements [29, 38]. Moreover, these measurements have superior intra‐ and interobserver reliability [26]. It was therefore decided to use EOS‐3D to assess HKA measurements instead of 2D, as done in previous CAS‐rTKA studies. The superiority of EOS‐3D measurements to assess the HKA is a strength of this study, increasing the validity of results. The coronal and sagittal measurements were performed on 2D‐EOS images since these measurements cannot be taken with this software and are therefore comparable to other CAS‐rTKA studies. We did not perform sagittal measurements of the femoral component, as the evaluation of the femoral component is complicated due to the overlap of the prosthetic femoral condyles.

This study has some limitations. There is a discrepancy in the number of patients included in the intervention and control groups. We decided that alignment measurements performed now on patients operated before 2002 were not representative due to the use of a different knee revision system and different surgeons. Therefore, it was not possible to include more patients in the historical control group, and patients undergoing rTKA will be operated with CAS and included in the intervention group. Due to the smaller size of the control group compared to the intervention group, the dispersion measures between the two groups could be expected to be unequal. This could lead to a lack of statistical power in the analysis. However, patient demographics and characteristics were equally distributed and comparable, regardless of the group size difference.

In some cases, rotational CT measurements were not possible due to scattering, difficulty in identification of the tuberosity or epicondyles and/or bone loss. Due to the low number of measurements and no gold standard to perform the measurements, the validity of this technique could be questioned. However, no better alternative for femoral and tibial rotation measurements exists yet, and measurements based on CT scan are still standard daily practice. Whether CAS leads to better functional results or improved prosthesis survival is still unclear as this was not the scope of this study and could be a valuable topic for future studies. Including patient satisfaction scores and long‐term results in the analysis can yield better insight into the validity of mechanical alignment and the influence of CAS‐rTKA on functional outcomes and survival. Nevertheless, as the literature on CAS‐rTKA and its influence on rotational alignment is limited, this study makes a valuable contribution to the existing literature.

## CONCLUSION

No evidence was found supporting the hypothesis that using CAS during revision TKA leads to improved coronal, sagittal or rotational alignment of a knee prosthesis. No significant differences were found in number of outliers when using CAS, compared to the conventional control group. Therefore, we did not obtain any evidence that CAS is of additional value in rTKA in terms of prosthetic alignment.

## AUTHOR CONTRIBUTIONS

T.E. Alling collected part of the data, performed the statistical analyses and wrote the article. M.F. Conteh‐Meijer set up the design, performed alignment measurements, collected part of the data and reviewed the article. Alexander L. Boerboom set up the design, performed the surgeries and alignment measurements, and reviewed the article. M. Stevens set up the design and reviewed the article. I. H. F. Reininga set up the design, assisted with the statistical analyses and reviewed the article.

## CONFLICT OF INTEREST STATEMENT

The authors declare no conflict of interest.

## ETHICS STATEMENT

This single‐centre prospective study was approved by the University Medical Center Groningen Ethics Committee (METc 2012/220) and carried out in accordance with the ethical standards of the Declaration of Helsinki. Informed consent was given by each patient included in this study.

## Data Availability

The data sets used and analysed during the current study are available from the corresponding author upon reasonable request.
